# Data on the mixing of non-Newtonian fluids by a Rushton turbine in a cylindrical tank

**DOI:** 10.1016/j.dib.2016.08.023

**Published:** 2016-08-16

**Authors:** Akhilesh Khapre, Basudeb Munshi

**Affiliations:** Department of Chemical Engineering, National Institute of Technology, Rourkela, Odisha, India

**Keywords:** Non-Newtonian fluids, Rushton turbine, Mixing time, Mixing efficiency, Cylindrical tank

## Abstract

The paper focuses on the data collected from the mixing of shear thinning non-Newtonian fluids in a cylindrical tank by a Rushton turbine. The data presented are obtained by using Computational Fluid Dynamics (CFD) simulation of fluid flow field in the entire tank volume. The CFD validation data for this study is reported in the research article ‘Numerical investigation of hydrodynamic behavior of shear thinning fluids in stirred tank’ (Khapre and Munshi, 2015) [1]. The tracer injection method is used for the prediction of mixing time and mixing efficiency of a Rushton turbine impeller.

**Specifications Table**

**Table:** 

Subject area	*Chemical Engineering*
More specific subject area	*Fluid Mechanics*
Type of data	*Tables, figures*
How data was acquired	*By simulating whole 3D domain of baffled cylindrical tank*
Data format	*Analyzed*
Experimental factors	*Carboxymethyl cellulose and Xanthangum solution are used as a working shear thinning non-Newtonian fluids for simulation*
Experimental features	*For simulation, the Ansys 13 is used to solve the continuity, momentum and species transport equations. A known concentration of a tracer which has same physical property of working fluid is injected in tank for mixing time prediction. The sliding mesh approach is applied for calculation of the mixing time in tank. The strain and vorticity tensors are calculated from simulated flow field inside a tank.*
Data source location	*Rourkela, India*
Data accessibility	*With this article*

**Value of the data**

• The data help to predict the performance of a Rushton turbine in a cylindrical tank in terms of mixing efficiency.
• It provides significant information about the mixing time in the transition and turbulent flow zone.
• It is also useful to explore the nature of the fluid flow and dispersive mixing efficiency inside the tank.

## 1 Data

In this article, the data generated on mixing of shear thinning non-Newtonian fluids by a Rushton turbine in a cylindrical tank is reported. The data is obtained using CFD simulation of whole tank. The validation of this study is found in [1]. The data presented herein showed some significant information about the mixing time and dispersive mixing efficiency of a Rushton turbine. We include figures and tables containing quantitative and qualitative information on the mixing time and its dispersive efficiency.

## 2 Experimental design, materials and methods

### 2.1 Tank geometry studied

**Fig. 1:**
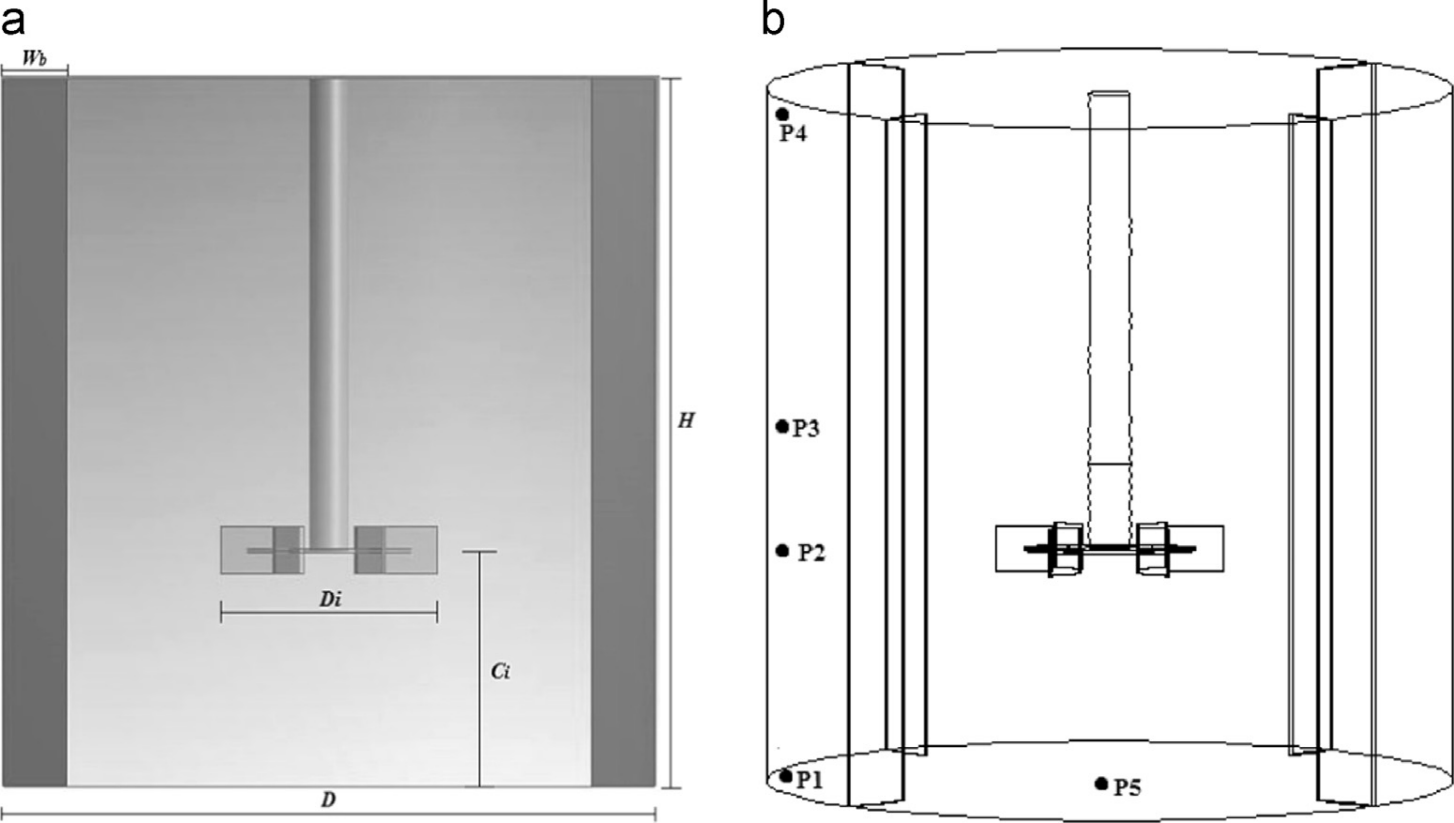
(a) Geometry of cylindrical tank, (b) the detail of locations in a tank: P_1_ (0.313, 0.05), P_2_ (0.313, 0.209), P_3_ (0.313, 0.3135), P_4_ (0.313, 0.62) and P_5_ (0.0, 0.0) (all measuring locations in (*r, z*) coordinate system).

The baffled cylindrical tank with a Rushton turbine to agitate non-Newtonian fluids is shown in Fig. 1a [2]. The detailed cylindrical tank dimensions are given in Table 1. Fig. 1b depicted the detail of locations in a tank where the tracer is injected (P_5_) and concentrations of the tracer is collected (P_1_–P_4_) with time to finding out the mixing time of the tank.

The rheological properties of the shear thinning non-Newtonian fluids are given in Table 2 [2], where *K* and *n* is the consistency index and flow behavior index, respectively.

### 2.2 Data obtained

#### 2.2.1 Mixing time

**Fig. 2:**
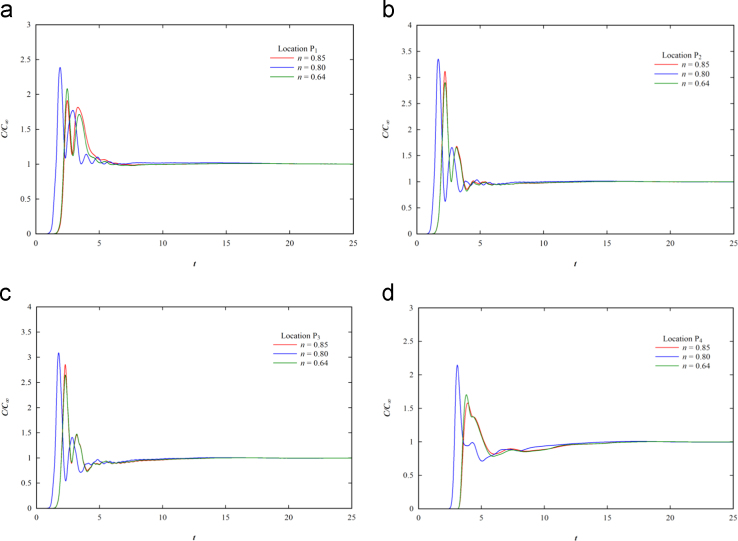
Response curve for the shear thinning fluid at 180 rpm impeller speed.

The mixing time in the tank is determined by plotting curves between the simulation predicted tracer concentration and time. The impeller rotations are kept constant at 180 rpm. The detailed experimental and CFD simulation method for estimating the mixing time is given in the literature [3], [4], respectively. A tracer is injected initially at midpoint of bottom surface of the tank, i.e., at location P_5_ and the concentrations at remaining cells are initialized to zero. Numerically the concentrations at locations P_1_–P_4_ are collected with time and the respective non-dimensional traction concentration distributions are presented in Fig. 2. The effect of impeller on fluid flow decreases as move from locations P_1_ to P_4_. The mixing time, *t*_*m*_, increases from the locations P_1_–P_4_ at all the impeller speeds irrespective of the flow behavior index (*n*). It is also seen that the impeller rotations has the huge effect on the mixing time.

**Fig. 3:**
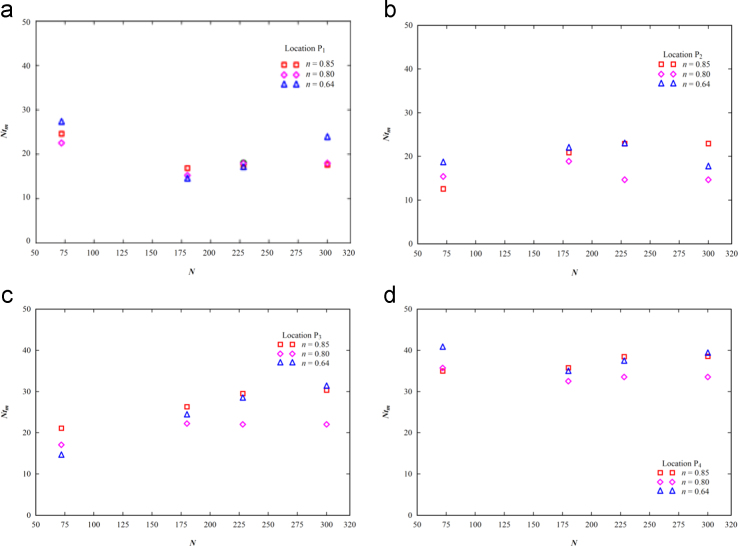
Distributions of the non-dimensional mixing time with impeller rotations for non-Newtonian fluids.

The calculated mixing time, *t*_*m*_ is multiplied by the impeller rotations, *N* to obtain the non-dimensional mixing time, *Nt*_*m*_. The chosen range of the impeller rotations make sure that the Reynolds number of fluid flow is either in transition or in the turbulent flow state. The distributions of the non-dimensional mixing time with the impeller speed are illustrated in Fig. 3. The figure shows that the nature of distribution of the *Nt*_*m*_ is dependent on the mixing study location and the distribution of the non-dimensional mixing time is scattered in nature.

#### 2.2.2 Dispersive mixing efficiency

The mixing efficiency in terms of dispersive mixing efficiency $\left(\alpha_{DME}\right)$ is defined as [5], [6]

(1) $$\alpha_{DME}=\frac{\left\|\dot{\gamma }\right\|}{\left\|\dot{\gamma }\right\|+\left\|\omega \right\|}$$

where $\dot{\gamma }$ is the rate of strain tensor and $\omega =1/2\left[\nabla v-{\left(\nabla v\right)}^{T}\right]$ is the vorticity tensor.

For $\alpha_{DME}=0$, a rotational flow and no effective mixing occur,

For $\alpha_{DME}=0.5$, a simple shear flow, and.

For $\alpha_{DME}=1$, a dispersive flow.

**Fig. 4:**
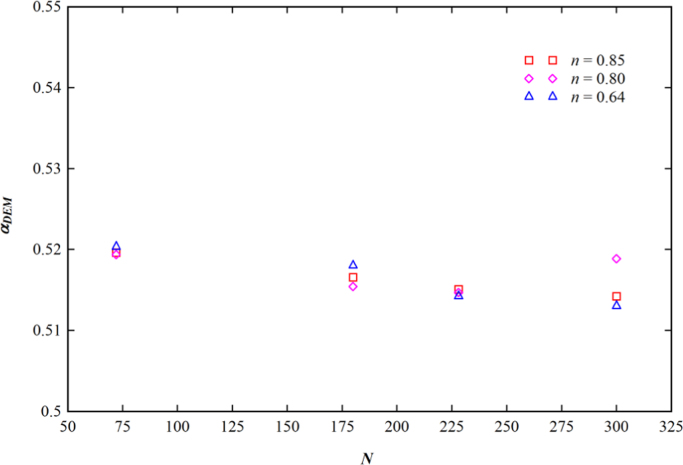
Distribution of the average dispersive mixing efficiency with impeller rotations.

Fig. 4 illustrates the distribution of $\alpha_{DME}$ for the Rushton turbine impeller. The figure shows that the average $\alpha_{DME}$ is little more than 0.5 for all impeller rotations and flow behavior indexes. Thus, the flow inside the stirred tank is a simple shear flow.

## Figures and Tables

**Table 1:** Tank dimensions.

*D* (m)	*H* (m)	*D*_*i*_ (m)	*C*_*i*_ (m)	*W*_*b*_ (m)
0.627	0.627	0.209	0.209	0.06

**Table 2:** Rheological properties of non-Newtonian Fluids.

Solution	*K* (kg s^*n*−2^ m^−1^)	*n* (−)
Carboxymethyl cellulose	0.0132	0.85
Xanthangum-1	0.0095	0.80
Xanthangum-2	0.034	0.64
